# Supplementary material for: The Medical Segmentation Decathlon
Source: Nat Commun. 2022 Jul 15;13:4128. doi: 10.1038/s41467-022-30695-9 (PMC9287542; doi:10.1038/s41467-022-30695-9)
Supplement: Supplementary file 1 — Supplementary Information [file 41467_2022_30695_MOESM1_ESM.pdf]

The Medical Segmentation Decathlon – Supplementary Information

Antonelli/Reinke et al.

Table of Contents

Supplementary Methods
- Supplementary Methods 1: Challenge organization 2
- Supplementary Methods 2: Method details 4

Supplementary Tables
- Supplementary Table 1: Details of the participant's methods. 8
- Supplementary Tables 2-11: Mean DSC values for all participating teams for all tasks. 9

Supplementary Figures
- Supplementary Figures 1-2: Dot- and box-plots of the NSD values of all participating algorithms. 19
- Supplementary Figures 3-12: Line plots visualizing rankings robustness across four different ranking methods for all tasks. 21
- Supplementary Figure 13: Stacked frequency plot showing the achieved ranks of the participating algorithms over 1,000 bootstrap datasets for all tasks for the DSC. 31
- Supplementary Figure 14: Dot- and box-plot of the mean DSC values computed for each task and target ROI for all algorithms in the 2018 MSD and live-decathlon challenges. 32

References 33

Supplementary Methods

Supplementary Methods 1: Challenge organization

The MSD challenge was organized in the scope of MICCAI 2018, held in Granada, Spain. It was organized by M. Jorge Cardoso (King's College London), Amber Simpson (Memorial Sloan Kettering Cancer Center), Olaf Ronneberger (Google Deep mind), Bjoern Menze (Technische Universität München), Bram van Ginneken (Radboud University Medical Center), Bennett Landman (Vanderbilt University), Geert Litjens (Radboud University Medical Center), Keyvan Farahani (National Institutes of Health), Ronald M. Summers (National Institutes of Health Clinical Center), Lena Maier-Hein (DKFZ German Cancer Research Center), Annette Kopp-Schneider (DKFZ German Cancer Research Center), Spyridon Bakas (CBICA, University of Pennsylvania) and Michela Antonelli (King's College London).

The challenge was organized as an open call event, i.e. after the challenge event at MICCAI, the challenge still accepted and evaluated submissions. For the submission itself, the grand-challenge.org platform was used (https://decathlon-10.grand-challenge.org), whereas all other information was given on a separated website (http://medicaldecathlon.com).

The participation policies of the MSD allowed only fully automatic methods without task-specific manual parameter settings to submit. In addition, there was no restriction in using other data sources to pre-train the individual methods, as long as that data was not modified per task. Only one team was allowed per research lab, as to avoid bypassing submission count restrictions. The first runner method of each phase, and the runner up of the mystery phase all received an NVIDIA Titan V prize. Ranking and results on each data set for each method were announced publicly at the challenge event at MICCAI and the post-challenge leaderboard is also publicly available. Finally, we asked the participating teams to fill out a form with details about their methods. All team members replying to the survey were listed as co-authors of the paper. Participants were allowed to publish their methods independently from the challenge paper.

All teams were asked to submit the results of the development phase as a compressed archive to the grand-challenge.org platform. A fully automated validation script was run for each submission immediately after submission and results were published on the development phase leaderboard. Each team was allowed one submission per day to partially mitigate overfitting. The last submission of each team by the development phase deadline was used for validation. Teams were then asked to submit details of their methods prior to being given access to the mystery phase data. The submission deadline for the mystery phase results was set to two weeks after the mystery phase data release, also in compressed archive form. Only a single valid submission was

accepted for each the mystery phase participant, and results for the mystery phase were only revealed at the public challenge event during MICCAI 2018, in Granada, Spain.

The implementation of the metrics used in the challenge, namely the DSC and NSD, were provided as a Python Notebook (http://medicaldecathlon.com/files/Surface_distance_based_measures.ipynb) by the challenge organizers, prior to the challenge deadlines. The algorithms for statistical validation were also provided.

As some of the participating teams were working under intellectual property restrictions, it was decided that public code availability was not mandatory for participation as to maximize participation; participants were, however, encouraged to make their code available to the public.

The challenge was organized without specific funding, and mostly via in-kind time contributions of its organizers. The challenge was sponsored by NVIDIA, who provided the graphics processing unit (GPU) cards as awards (approximate value of $7,500), Google DeepMind, who provided an in-kind implementation of the NSD metric, and by RSIP Vision, who provided media support and challenge dissemination. None of the sponsors had any influence in the organization of the challenge, nor were they given any form of privileged access to either the data or any other type of information.

Only two of the organizers, both from KCL, had access to all test cases; namely M. Jorge Cardoso and Michela Antonelli. The KCL organizers committed to not participate in the challenge. Only two copies of the full test data currently exist (beyond the data providers of each independent task), one at KCL's servers as a backup, and one on the grand-challenge.org validation server.

Supplementary Methods 2: Method details

In the following, we provide details for the remaining teams that submitted a description of their methods. Note that the methods from the top three teams are presented in Section 2.2 (Method description of top three algorithm) of the main paper.

AI-MED

The team used the QuickNAT with added Conditional Random Fields (CRF) [1].

The DSC loss was combined with the cross entropy loss and the SGD optimizer was used. No data augmentation or ensembling techniques were employed.

BCVuniandes

The team employed DeepMedic [2] as base architecture by using two identical parallel pathways with multi-scale analysis. Each pathway had four stages and all the intermediate outputs were resized and concatenated to be processed by two fully connected layers. The team did not apply augmentation or ensembling techniques, but used a softmax cross-entropy loss and the Adam optimizer.

CerebriuDIKU

The method used the standard 2D U-Net architecture with added batch normalization layers intervening each double-convolution- and up-convolution block and nearest neighbour up-sampling [3].

Two augmentations were applied, namely non-linear deformation and multi-planar sampling of 2D image planes. Furthermore, the cross entropy loss and the Adam optimizer were applied. Averaging multiple runs of the same architecture was the used ensembling strategy.

The key aspect of the method was the multi-planar training that allowed for a huge number of anatomically relevant images to be augmented during training, exposing the model to a broader representation of the 3D image volume while maintaining the parameter (and computational) efficiency of 2D kernels. Optimizing over multiple planes increased the complexity of the target function making overfitting less likely but maintained performance by doing so through the exposure of the model to additional data. The model learned to segment the target as seen from multiple views and can therefore be used to predict the same target multiple times.

Lesswire1

The team used a U-Net architecture with concatenated lower resolution features before upscaling and the transpose convolution for upscaling.

Test-time augmentation was applied and a depth-wise cross-entropy was the loss function which consisted of a weighted sum of depth-wise cross entropy and L2 norm. Adam was used and no ensembling techniques were applied.

The key idea of the model was that it was designed to operate on any volume regardless of the number of slices. No assumption was made to limit the model to a specific organ. Losses associated with anomalies were weighted based on the training data adaptively so that the model loss was not specific to a certain organ.

LfB

The team modified the U-Net architecture with residual connections per block, deep supervision (multi-level generation of segmentations), and instance normalization over batch normalization [4].

The following augmentation strategies were used: affine transformation, non-linear deformation, noise addition, rotation, and random crop; moreover, if anisotropy, elastic deformation field was scaled to yield isotropic world coordinate scaling. DSC loss and Adam were used. No ensembling strategy was applied.

The key point of the method was to do as little task-specific engineering as possible (i.e. during training only resampled to median voxel spacing, got median shape, and inferred model geometry/architecture from median shape; for inference, resampled to median voxel spacing, applied patch-wise prediction, and resampled to original voxel spacing).

LS Wang's

The team proposed a modification of the U-Net, more specifically a Nested Dilation Network (NDN) which was applied to multiple segmentation tasks and multiple modalities. The Residual Blocks Nested were designed with dilations (RnD Blocks) which catch larger receptive field in the first few layers to boost shallow semantic information [5].

The following augmentation strategies were applied: affine transformations, non-linear deformation, noise addition, histogram transformation, geometric left-right flip, and random crop.

The team used a modification of focal loss and the Adam optimizer and applied averaging multiple runs of the same architecture as ensambling strategy.

The unique configuration was the key to cope with the ten different tasks.

Lupin

To take advantage of the ability of the U-Net in combining high-level features and low-level features, the team added deep-supervision during training on decoding path.

Affine transformation, noise addition, and random crop were applied as augmentation techniques. Focal loss and Adam were used. No ensembling strategy was employed.

MIMI

U-Net was the base network architecture of this team, which was used with the following additional components: 1) residual Block: skip connections inside convolutional blocks; 2) auxiliary losses for deep supervision; 3) leaky ReLU; 4) dropout inside decoders. The team applied affine transformations, geometric left-right flip, and random crop as augmentation strategy. DSC loss and the Adam optimizer were employed. They average multiple runs of the same architecture as ensambling strategy.

Using Multi-Task Learning (MTL) was the key point of this approach. Representations among related tasks were shared and a better generalization achieved.

Jiafucang

A modified version of V-Net was used. In particular, they used a two-level V-Net model with batch normalization after each convolution in the second layer and set the number of feature maps as 32 after the first convolution. The input size was still 128×128×64 except for those tasks who have ROI larger than 128 in x- or y-axis, otherwise a size of 160×160×64 was used for the memory limitation.

Two augmentation techniques were applied, namely non-linear deformation and histogram transformation.

DSC loss and weighted softmax were used. SGD was the optimizer. No ensambling techniques were applied.

The key aspect of the method was using a two-stage coarse-to-fine method that is a general approach for medical volume segmentation.

They proposed an automatic ROI extraction technique based on the initial coarse segmentation in the first level. The input size of the V-Net in the second level had two types which were decided according to the extracted ROI.

VST

The team used different models depending on the task. More specifically, they used a 2.5D U-Net, a 3D CNN and a 3D U-Net for detection, classification, and segmentation, respectively.

2.5D U-Net considered a total of 5 slices adjacent in an axial direction and output the segmentation probability at the central position among the 5 slices. 3D CNN considered 3D voxels and classified whether the target objects included the voxel or not.

Affine transformations (rotation, translation, scale) were applied as augmentation techniques. The loss function was different for each model: weighted cross entropy for the 2.5D U-Net, cross entropy for 3D CNN, and weighted cross entropy for 3D U-Net. The optimizer was SGD for 3D CNN and Adam for the other models. Multiple-architecture weighted averaging was used as ensembling technique.

Whale

An ensemble of both 3D and 2D U-Nets was used, in which the 3D U-Net was shallow while the 2D U-Net was much deeper. Geometric left-right flip and random crop were applied as augmentation techniques. The team employed cross entropy loss with larger weights to minority classes and the SGD optimizer.

Multiple-architecture non-weighted averaging was the ensembling strategy.

The method relied on the use of U-Net which is very general and its effectiveness has been demonstrated on many tasks.

Supplementary Tables

Supplementary Table 1: Details of the participant's methods who provided full algorithmic information (n=14 teams).

Team	Base Architecture & Modifications	Augmentation Strategy	Loss Function	Optimizer Training	Pre-processing	Post-processing	Ensembling strategy
nnU-Net[1]	U-Net – leaky ReLU, instance normalization, strided convolutions	Affine, non-linear, intensity, flipping along all axes, random crop	DSC loss cross entropy loss	Adam (learning rate=3e-4, weight decay=3e-5)	1,000 epochs with early stopping & Intensity normalization, padding/cropping, rescaling interpolation	Segmentation region removal	Training set cross-validation model selection
NVDLMED	ResNet, anisotropic 3D kernels	Affine, left-right flip, random crop	DSC loss	SGD (learning rate =7e-3, weight decay=3e-5, momentum=0.9, batch size=8) 40,000 epochs	Intensity normalization, cropping, rescaling interpolation	Region removal	Ensemble three trained views
K.A.V.athlon	U-Net, V-Net Squeeze-and-Excitation	Affine, noise, left-right flip, random crop, blur	DSC loss	Adam (learning rate =1e-4)	Intensity normalization, rescaling interpolation	Segmentation region removal	/
LS Wang's Group[2]	U-Net residual blocks nested with dilations	Affine, histogram, left-right flip, random crop	Modified focal loss	Adam (learning rate=1e-4), 90,000 epochs	Padding/cropping, rescaling interpolation	/	Averaging multiple runs
MIMI	U-Net skip connections inside convolutional blocks, leaky ReLU,	Affine, noise, left-right flip, random crop	DSC loss	Adam (learning rate=0.5e-4)	Intensity normalization, rescaling interpolation	Intensity-based region removal	Averaging multiple runs
Cerebriu DIKU[3]	U-Net, batch normalization layers	Non-linear, multi-planar sampling	Cross entropy loss	Adam (learning rate=5e-5, beta_1=0.9, beta_2=0.999, epsilon=1e-8)	Intensity normalization, multi-planar sampling	/	Averaging multiple runs
Whale	U-Net 3D/2D	Left-right flip, random crop	Cross entropy loss	Adam (learning rate=5e-5, beta_1=0.9, beta_2=0.999, epsilon=1e-8)	Padding/cropping	Segmentation region removal and masking	Multiple-architecture non-weighted averaging
Lupin[4]	U-Net	Affine, noise, random crop	Focal loss	Adam (learning rate=1e-4)	Intensity normalization	/	/
Jiafucang	V-Net batch normalization	Non-linear, histogram	DSC and weighted softmax loss	SGD (learning rate=1e-4/0.5e-4)	Intensity normalization, padding/cropping, rescaling interpolation	Segmentation region removal	/
LfB	U-Net residual connections, instance normalization	Affine, noise, non-linear, random crop	DSC loss	Adam (learning rate=0.5e-4), 30,000 epochs	Padding/cropping, rescaling interpolation, resampling to the median voxel-spacing	/	/
VST	U-Net 2.5D/3D	Affine	Cross entropy loss	Adam and SGD (learning rate=0.1, momentum=0.9)	Intensity normalization, padding/cropping, rescaling interpolation	Segmentation region removal, intensity-based	Multiple-architecture weighted averaging
AI-MED	QuickNAT CRF	/	DSC loss, cross entropy loss	SGD (learning rate=0.01, batch size =4, momentum=0.9), 20 epochs	Padding/cropping	Conditional random field/ graph-cuts	/
Lesswire1[5]	U-Net	Test-time augmentation	Depth-wise cross entropy loss	Adam (learning rate=1e-4)	Intensity normalization	/	/
BCV Uniandes	DeepMedic[6]	/	Cross entropy loss	Adam (learning rate=1e-4)	Intensity normalization, rescaling interpolation	Region removal	/

URLs to source code:

[1] https://github.com/MIC-DKFZ/nnUNet

[2] https://github.com/wangshuxinxinxin/MSD

[3] https://github.com/perslev/MultiPlanarUNet

[4] https://github.com/xzwthu/SegUNet

[5] https://gitlab.com/lesswire1/Medical_Segmentation

[6] https://github.com/BCV-Uniandes/ROG

Supplementary Table 2: Mean Dice Similarity Coefficient (DSC) values for all 19 participating teams for all tasks (edema, non-enhancing tumor, and enhancing tumor) of the brain data set (the development phase).

Algorithm	Edema	Non-enhancing tumor	Enhancing tumor
nnU-Net	0.68	0.48	0.68
NVDLMED	0.68	0.45	0.68
K.A.V.athlon	0.66	0.47	0.67
LS Wang's Group	0.68	0.46	0.66
MIMI	0.65	0.44	0.66
CerebriuDIKU	0.70	0.43	0.67
Whale	0.64	0.30	0.23
UBIlearn	0.65	0.37	0.62
Lupin	0.66	0.42	0.64
Jiafucang	0.33	0.27	0.31
LfB	0.60	0.44	0.62
A-REUMI01	0.64	0.40	0.64
VST	0.54	0.30	0.63
AI-Med	0.64	0.35	0.61
Lesswire1	0.63	0.41	0.58
BUT	0.64	0.35	0.62
RegionTec	0.42	0.35	0.55
BCVuniandes	0.69	0.43	0.65
EdwardMa12593	0.37	0.01	0.18
**Median**	0.64	0.41	0.63

Supplementary Table 3: Mean Dice Similarity Coefficient (DSC) values for all 19 participating teams for all tasks (left atrium) of the heart data set (the development phase).

Algorithm	Left Atrium
nnU-Net	0.93
NVDLMED	0.92
K.A.V.athlon	0.92
LS Wang's Group	0.90
MIMI	0.90
CerebriuDIKU	0.89
Whale	0.89
UBIlearn	0.91
Lupin	0.92
Jiafucang	0.88
LfB	0.91
A-REUMI01	0.90
VST	0.89
AI-Med	0.76
Lesswire1	0.72
BUT	0.76
RegionTec	0.63
BCVuniandes	0.80
EdwardMa12593	0.73
**Median**	0.89

Supplementary Table 4: Mean Dice Similarity Coefficient (DSC) values for all 19 participating teams for all tasks (anterior, posterior) of the hippocampus data set (the development phase).

Algorithm	Anterior	Posterior
nnU-Net	0.90	0.89
NVDLMED	0.88	0.87
K.A.V.athlon	0.90	0.89
LS Wang's Group	0.90	0.89
MIMI	0.89	0.88
CerebriuDIKU	0.90	0.88
Whale	0.86	0.85
UBIlearn	0.89	0.85
Lupin	0.90	0.88
Jiafucang	0.88	0.86
LfB	0.79	0.84
A-REUMI01	0.88	0.86
VST	0.86	0.87
AI-Med	0.83	0.73
Lesswire1	0.80	0.77
BUT	0.83	0.83
RegionTec	0.72	0.70
BCVuniandes	0.89	0.88
EdwardMa12593	0.87	0.87
**Median**	0.88	0.87

Supplementary Table 5: Mean Dice Similarity Coefficient (DSC) values for all 19 participating teams for all tasks (liver, cancer) of the liver data set (the development phase).

Algorithm	Liver	Cancer
nnU-Net	0.95	0.74
NVDLMED	0.95	0.71
K.A.V.athlon	0.95	0.62
LS Wang's Group	0.94	0.55
MIMI	0.94	0.60
CerebriuDIKU	0.94	0.57
Whale	0.94	0.62
UBIlearn	0.94	0.50
Lupin	0.95	0.61
Jiafucang	0.84	0.29
LfB	0.90	0.46
A-REUMI01	0.93	0.43
VST	0.93	0.54
AI-Med	0.91	0.51
Lesswire1	0.85	0.48
BUT	0.94	0.60
RegionTec	0.91	0.32
BCVuniandes	0.87	0.44
EdwardMa12593	0.81	0.15
**Median**	0.94	0.54

Supplementary Table 6: Mean Dice Similarity Coefficient (DSC) values for all 19 participating teams for all tasks (tumor) of the lung data set (the development phase).

**Algorithm**	**Tumor**
nnU-Net	0.69
NVDLMED	0.52
K.A.V.athlon	0.61
LS Wang's Group	0.55
MIMI	0.55
CerebriuDIKU	0.59
Whale	0.51
UBIlearn	0.51
Lupin	0.55
Jiafucang	0.55
LfB	0.47
A-REUMI01	0.45
VST	0.48
AI-Med	0.20
Lesswire1	0.18
BUT	0.33
RegionTec	0.00
BCVuniandes	0.42
EdwardMa12593	0.08
**Median**	0.51

Supplementary Table 7: Mean Dice Similarity Coefficient (DSC) values for all 19 participating teams for all tasks (pancreas, tumor mass) of the pancreas data set (the development phase).

Algorithm	Pancreas	Tumor mass
nnU-Net	0.80	0.52
NVDLMED	0.78	0.38
K.A.V.athlon	0.75	0.43
LS Wang's Group	0.71	0.26
MIMI	0.70	0.26
CerebriuDIKU	0.71	0.25
Whale	0.66	0.28
UBIlearn	0.69	0.18
Lupin	0.76	0.21
Jiafucang	0.56	0.20
LfB	0.59	0.28
A-REUMI01	0.65	0.20
VST	0.71	0.37
AI-Med	0.48	0.04
Lesswire1	0.55	0.07
BUT	0.73	0.21
RegionTec	0.61	0.07
BCVuniandes	0.56	0.15
EdwardMa12593	0.50	0.00
**Median**	0.69	0.21

Supplementary Table 8: Mean Dice Similarity Coefficient (DSC) values for all 19 participating teams for all tasks (peripheral zone (PZ), transition zone (TZ)) of the prostate data set (the development phase).

**Algorithm**	**PZ**	**TZ**
nnU-Net	0.76	0.90
NVDLMED	0.69	0.87
K.A.V.athlon	0.73	0.88
LS Wang's Group	0.71	0.85
MIMI	0.71	0.87
CerebriuDIKU	0.69	0.86
Whale	0.70	0.88
UBIlearn	0.67	0.84
Lupin	0.72	0.88
Jiafucang	0.70	0.84
LfB	0.58	0.82
A-REUMI01	0.67	0.86
VST	0.72	0.86
AI-Med	0.56	0.78
Lesswire1	0.52	0.78
BUT	0.65	0.85
RegionTec	0.34	0.64
BCVuniandes	0.69	0.86
EdwardMa12593	0.43	0.74
**Median**	0.69	0.86

Supplementary Table 9: Mean Dice Similarity Coefficient (DSC) values for all 19 participating teams for all tasks (cancer primaries) of the colon data set (the mystery phase).

Algorithm	Cancer primaries
nnU-Net	0.56
NVDLMED	0.56
K.A.V.athlon	0.36
LS Wang's Group	0.41
MIMI	0.29
CerebriuDIKU	0.28
Whale	0.18
UBIlearn	0.16
Lupin	0.09
Jiafucang	0.19
LfB	0.24
A-REUMI01	0.12
VST	0.15
AI-Med	0.11
Lesswire1	0.10
BUT	0.05
RegionTec	0.06
BCVuniandes	0.06
EdwardMa12593	0.06
**Median**	0.16

Supplementary Table 10: Mean Dice Similarity Coefficient (DSC) values for all 19 participating teams for all tasks (vessel, tumor) of the hepatic vessel data set (the mystery phase).

Algorithm	Vessel	Tumor
nnU-Net	0.63	0.69
NVDLMED	0.63	0.64
K.A.V.athlon	0.63	0.63
LS Wang's Group	0.55	0.64
MIMI	0.60	0.56
CerebriuDIKU	0.59	0.38
Whale	0.56	0.46
UBIlearn	0.59	0.37
Lupin	0.60	0.47
Jiafucang	0.51	0.37
LfB	0.55	0.35
A-REUMI01	0.56	0.39
VST	0.44	0.36
AI-Med	0.42	0.26
Lesswire1	0.44	0.31
BUT	0.44	0.39
RegionTec	0.42	0.20
BCVuniandes	0.14	0.32
EdwardMa12593	0.14	0.11
**Median**	0.55	0.38

Supplementary Table 11: Mean Dice Similarity Coefficient (DSC) values for all 19 participating teams for all tasks (spleen) of the spleen data set (the mystery phase).

Algorithm	Spleen
nnU-Net	0.96
NVDLMED	0.96
K.A.V.athlon	0.97
LS Wang's Group	0.96
MIMI	0.94
CerebriuDIKU	0.95
Whale	0.95
UBIlearn	0.95
Lupin	0.94
Jiafucang	0.93
LfB	0.83
A-REUMI01	0.92
VST	0.94
AI-Med	0.91
Lesswire1	0.86
BUT	0.89
RegionTec	0.92
BCVuniandes	0.82
EdwardMa12593	0.83
**Median**	0.94

Supplementary Figures

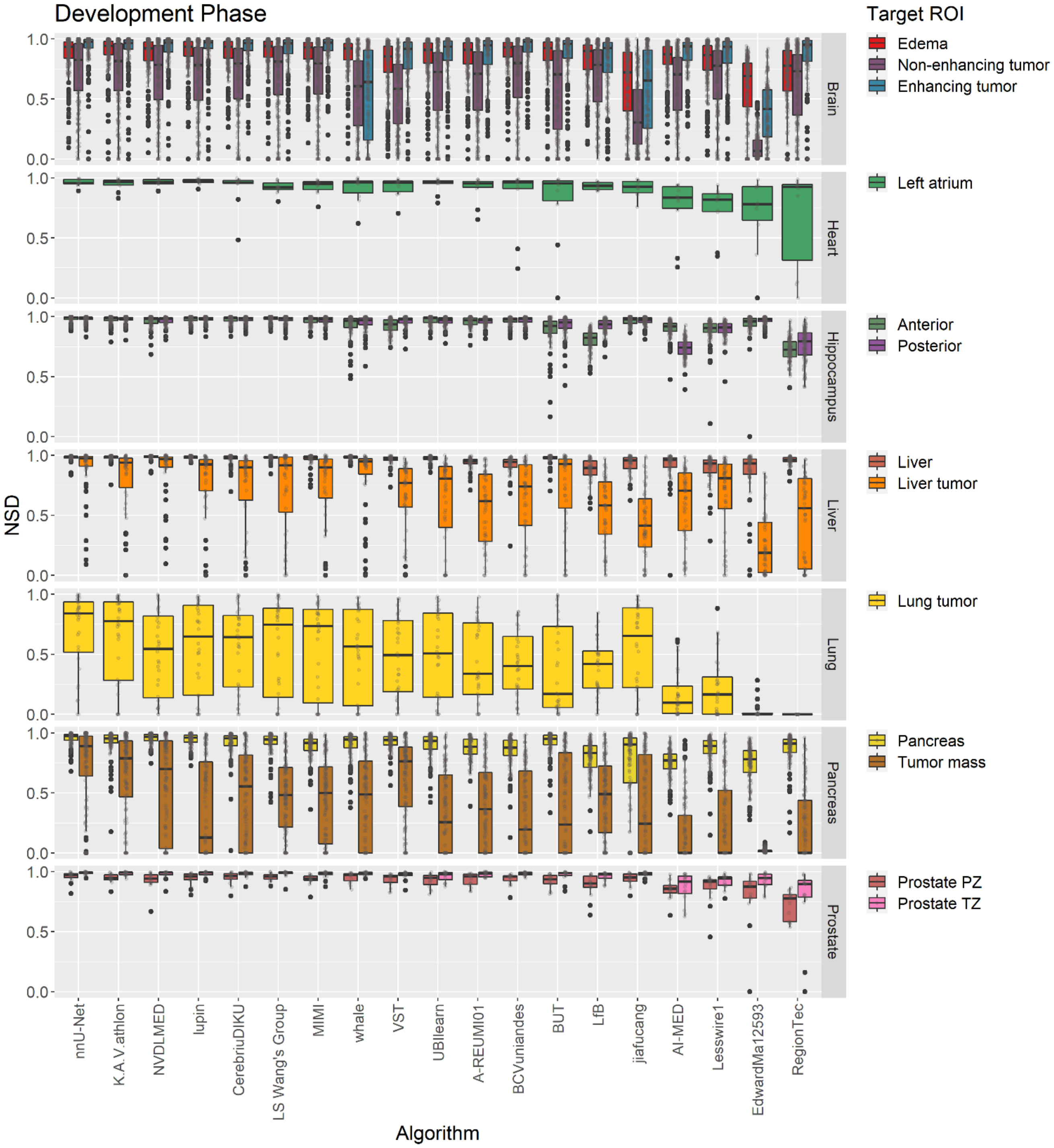

Supplementary Figure 1: Dot- and box-plots of the Normalized Surface Dice (NSD) values of all 19 participating algorithms for the seven tasks of the development phase, color-coded by the target regions. box-plots represent descriptive statistics over all test cases. The median value is shown by the black horizontal line within the box, the first and third quartiles as the lower and upper border of the box, respectively, and the 1.5 interquartile range by the vertical black lines. Outliers are shown as black circles. The raw NSD values are provided as gray circles. Used abbreviations: PZ – peripheral zone, TZ – transition zone.

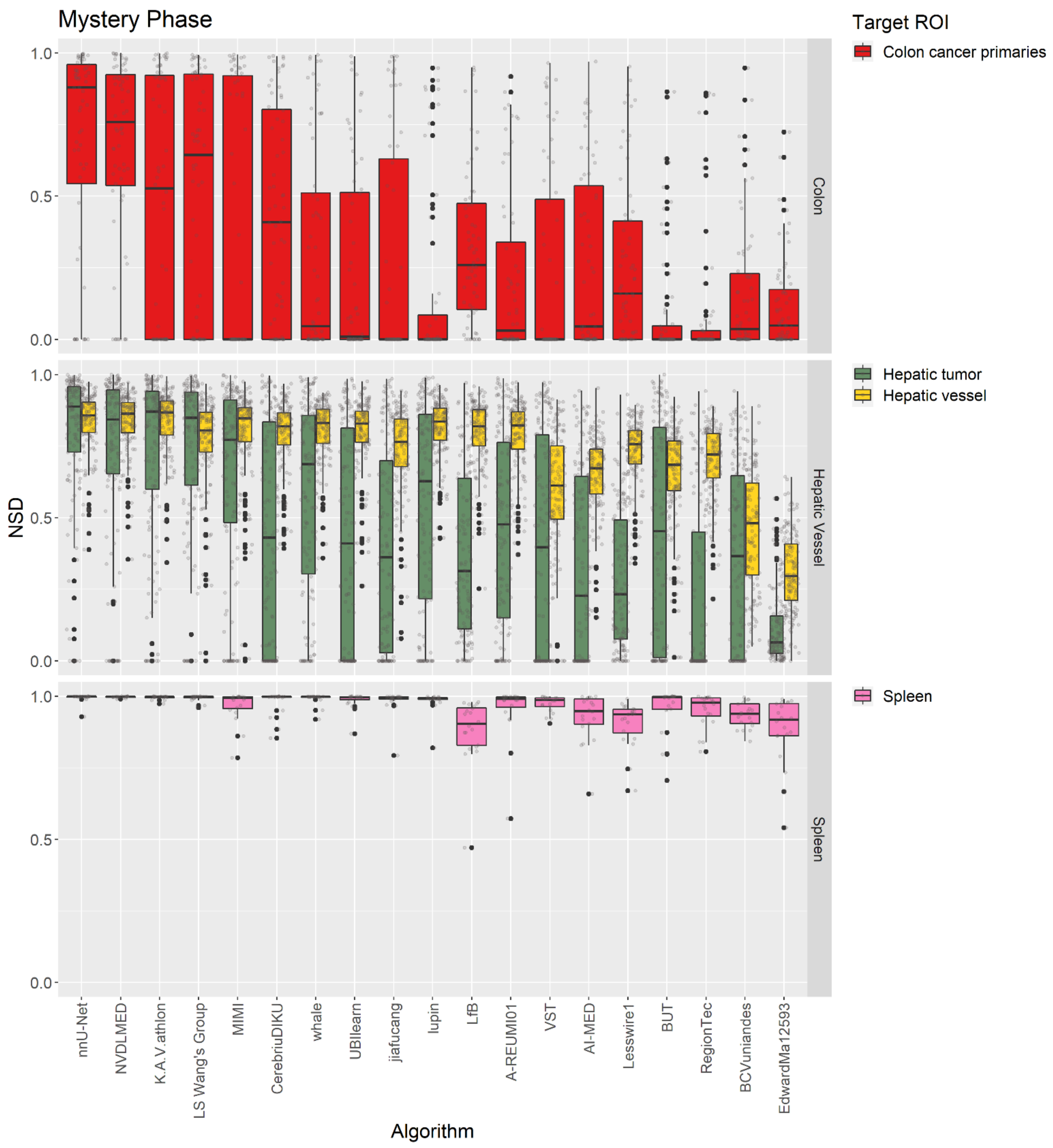

Supplementary Figure 2: Dot- and box-plots of the Normalized Surface Dice (NSD) values of all 19 participating algorithms for the three tasks of the mystery phase, color-coded by the target regions. box-plots represent descriptive statistics over all test cases. The median value is shown by the black horizontal line within the box, the first and third quartiles as the lower and upper border of the box, respectively, and the 1.5 interquartile range by the vertical black lines. Outliers are shown as black circles. The raw NSD values are provided as gray circles.

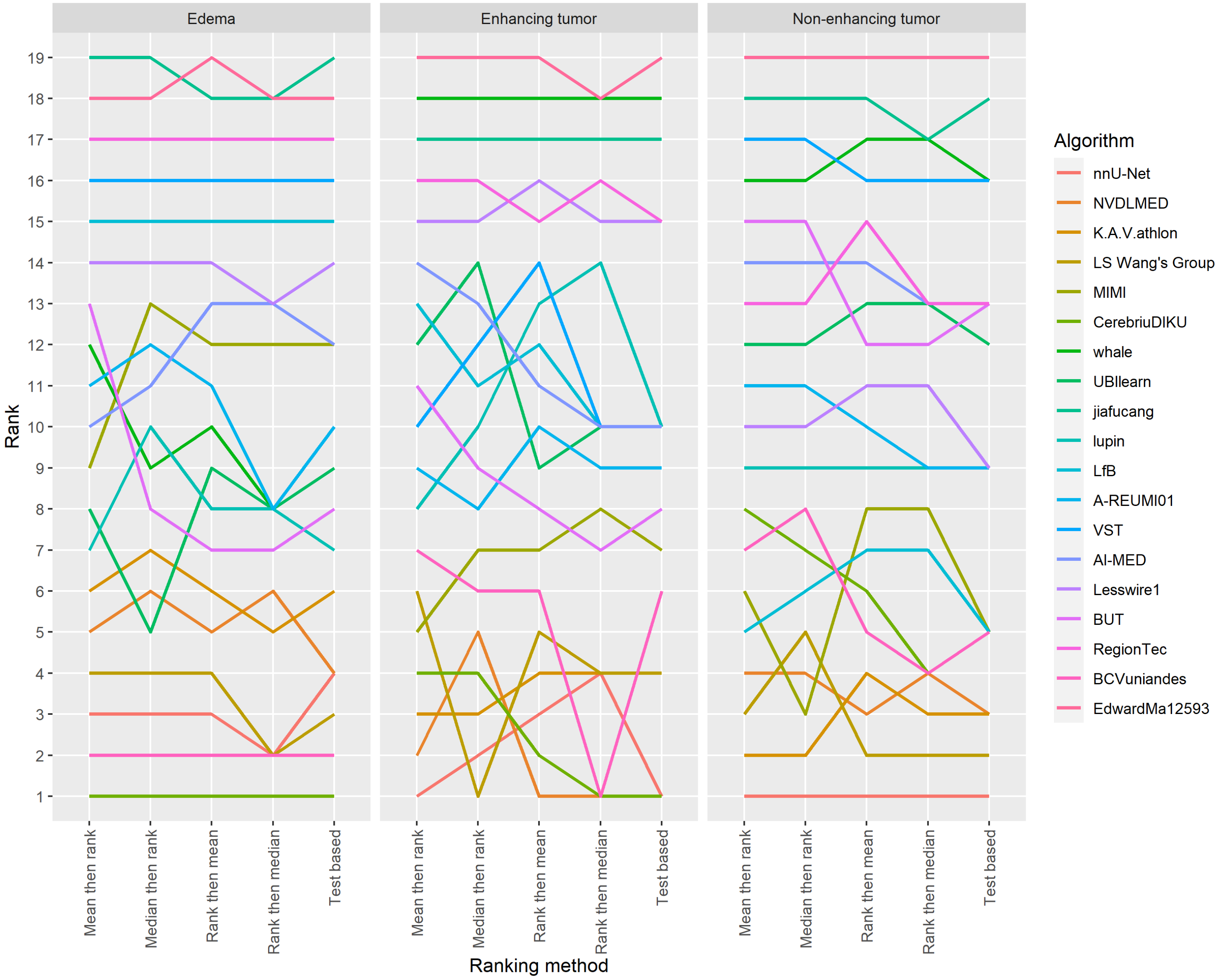

Supplementary Figure 3: Line plots visualizing rankings robustness across four different ranking methods for the brain task. Each of the 19 algorithms is represented by one colored line. For every ranking method encoded on the x-axis, the height of the line represents the corresponding rank. Horizontal lines indicate identical ranks for all methods under all ranking criteria.

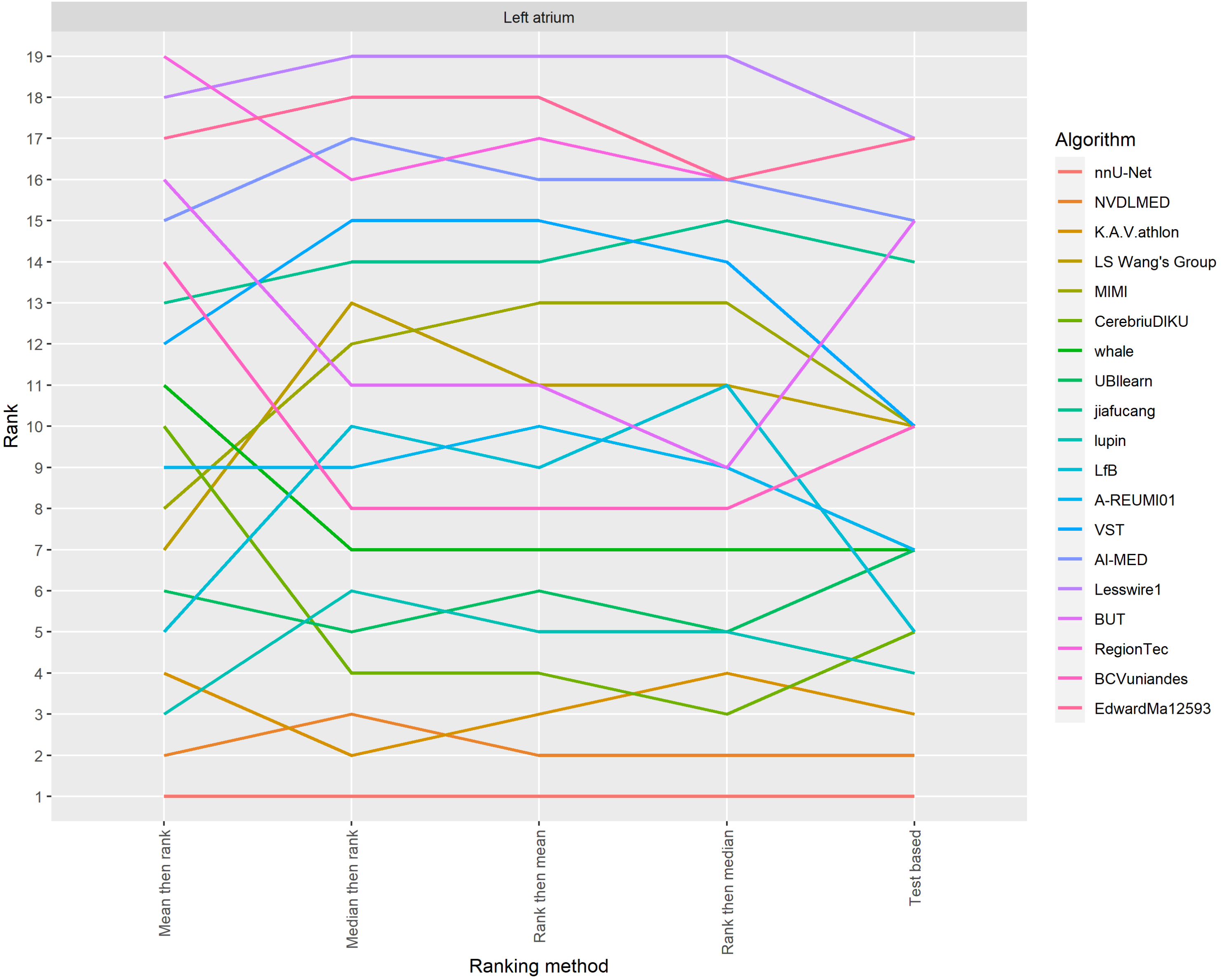

Supplementary Figure 4: Line plots visualizing rankings robustness across four different ranking methods for the heart task. Each of the 19 algorithms is represented by one colored line. For every ranking method encoded on the x-axis, the height of the line represents the corresponding rank. Horizontal lines indicate identical ranks for all methods under all ranking criteria.

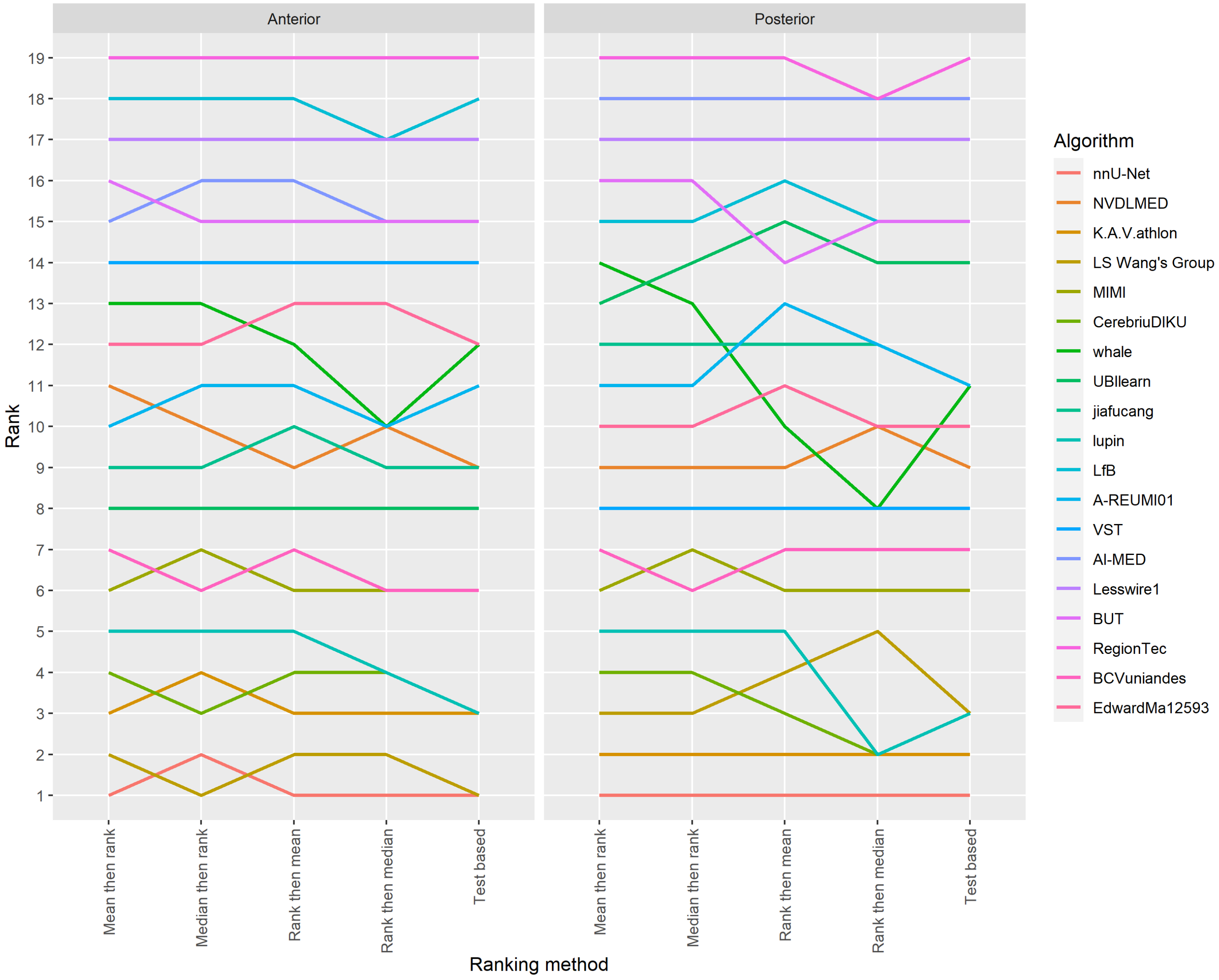

Supplementary Figure 5: Line plots visualizing rankings robustness across four different ranking methods for the hippocampus task. Each of the 19 algorithms is represented by one colored line. For every ranking method encoded on the x-axis, the height of the line represents the corresponding rank. Horizontal lines indicate identical ranks for all methods under all ranking criteria.

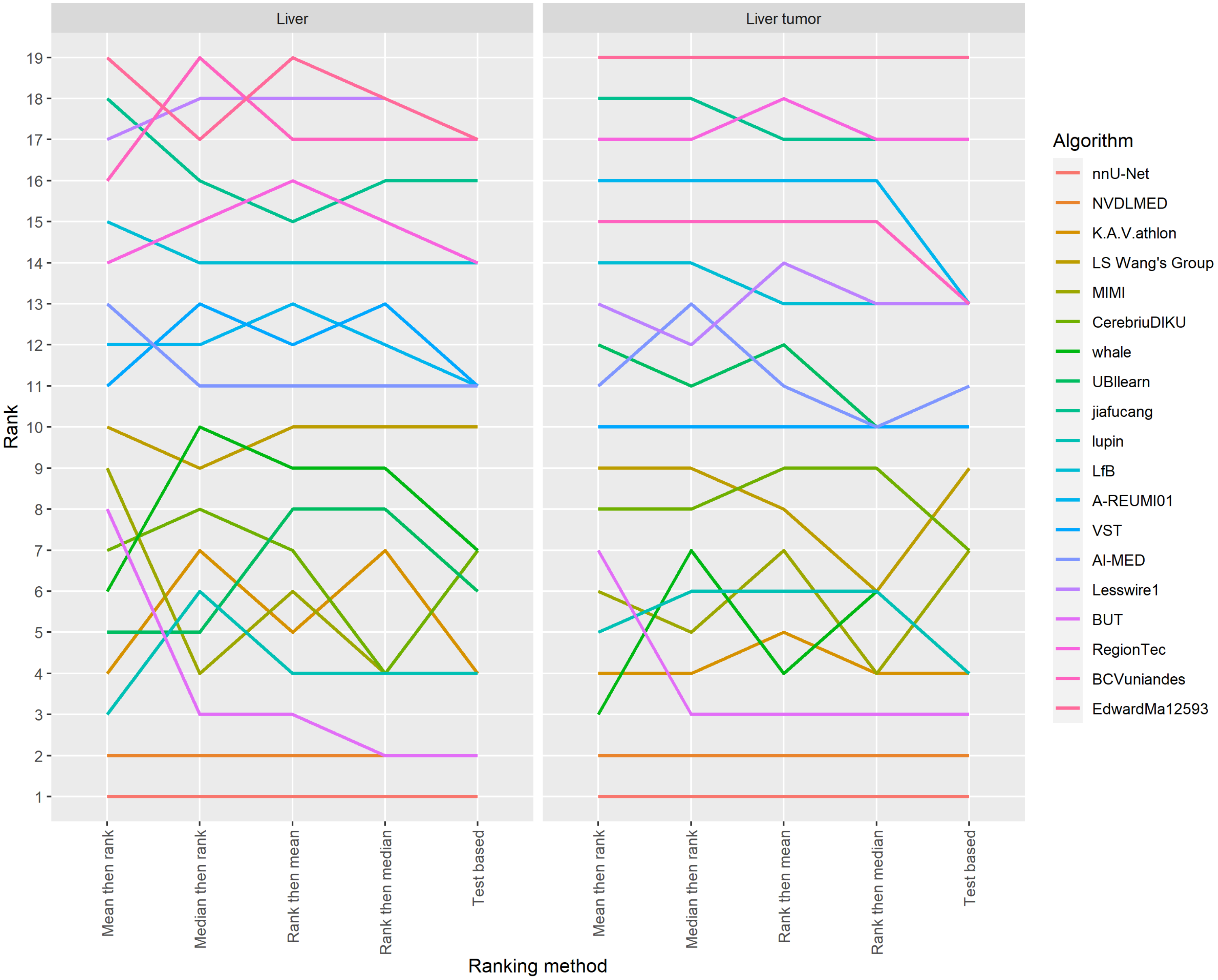

Supplementary Figure 6: Line plots visualizing rankings robustness across four different ranking methods for the liver task. Each of the 19 algorithms is represented by one colored line. For every ranking method encoded on the x-axis, the height of the line represents the corresponding rank. Horizontal lines indicate identical ranks for all methods under all ranking criteria.

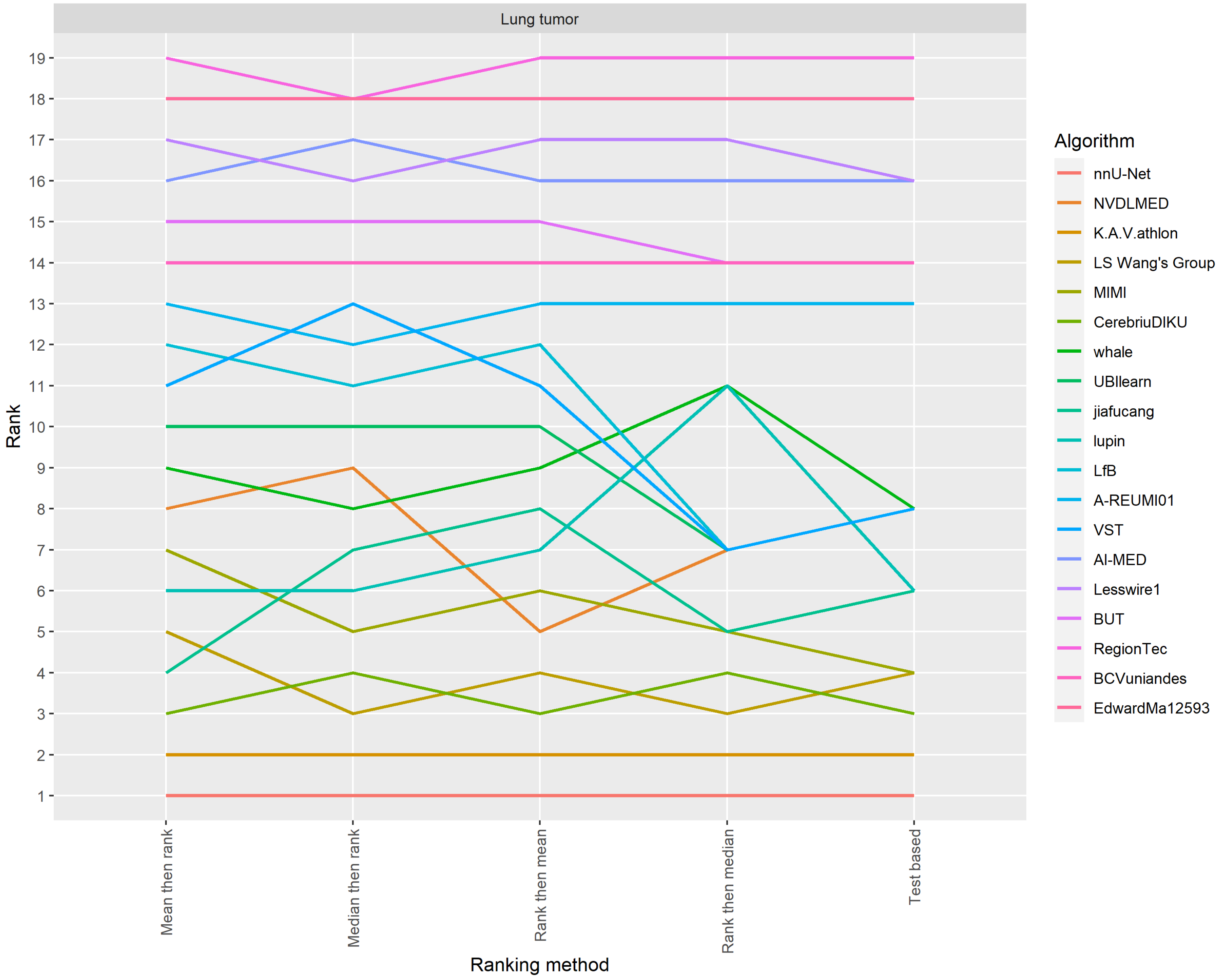

Supplementary Figure 7: Line plots visualizing rankings robustness across four different ranking methods for the lung task. Each of the 19 algorithms is represented by one colored line. For every ranking method encoded on the x-axis, the height of the line represents the corresponding rank. Horizontal lines indicate identical ranks for all methods under all ranking criteria.

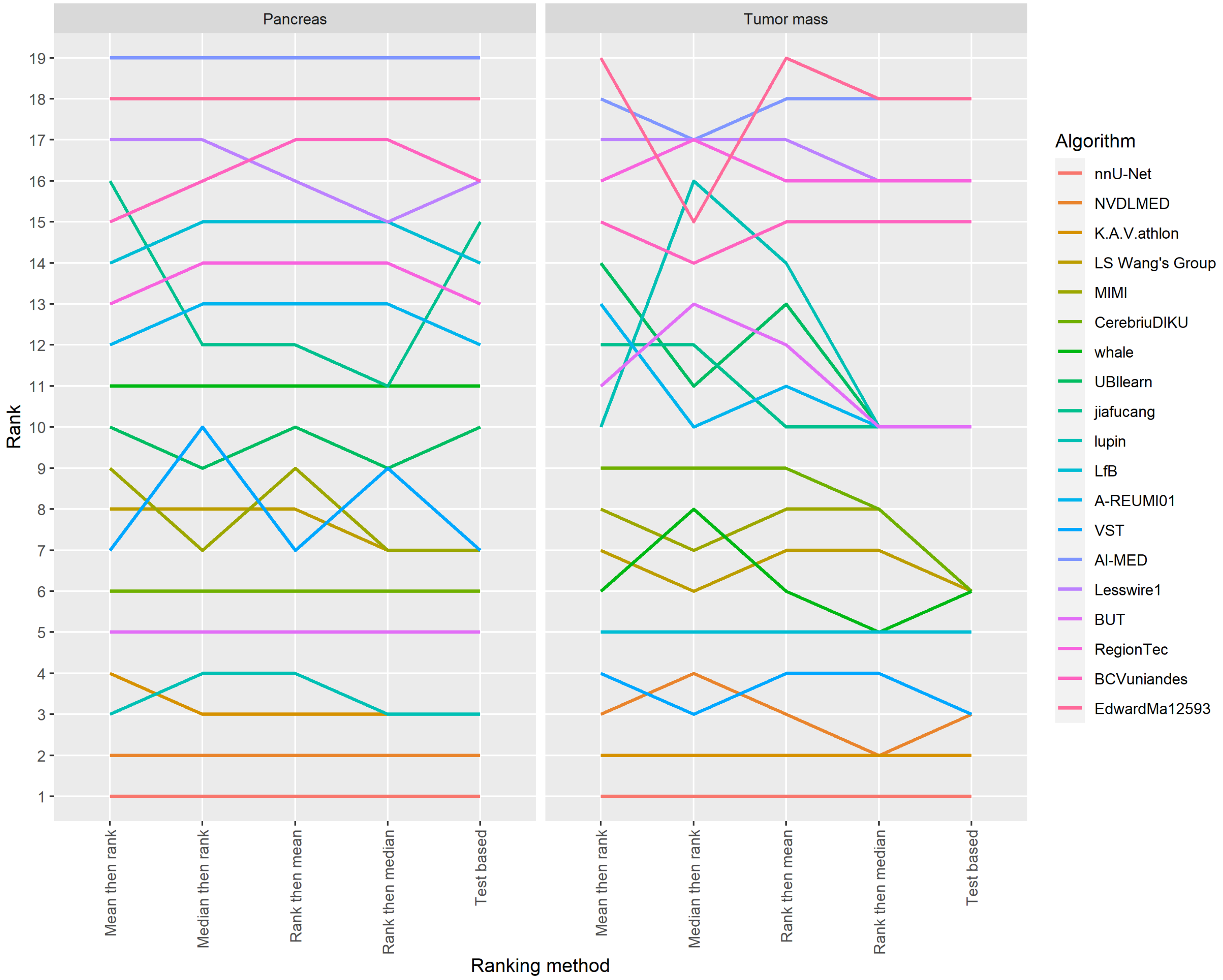

Supplementary Figure 8: Line plots visualizing rankings robustness across four different ranking methods for the lung task. Each of the 19 algorithms is represented by one colored line. For every ranking method encoded on the x-axis, the height of the line represents the corresponding rank. Horizontal lines indicate identical ranks for all methods under all ranking criteria.

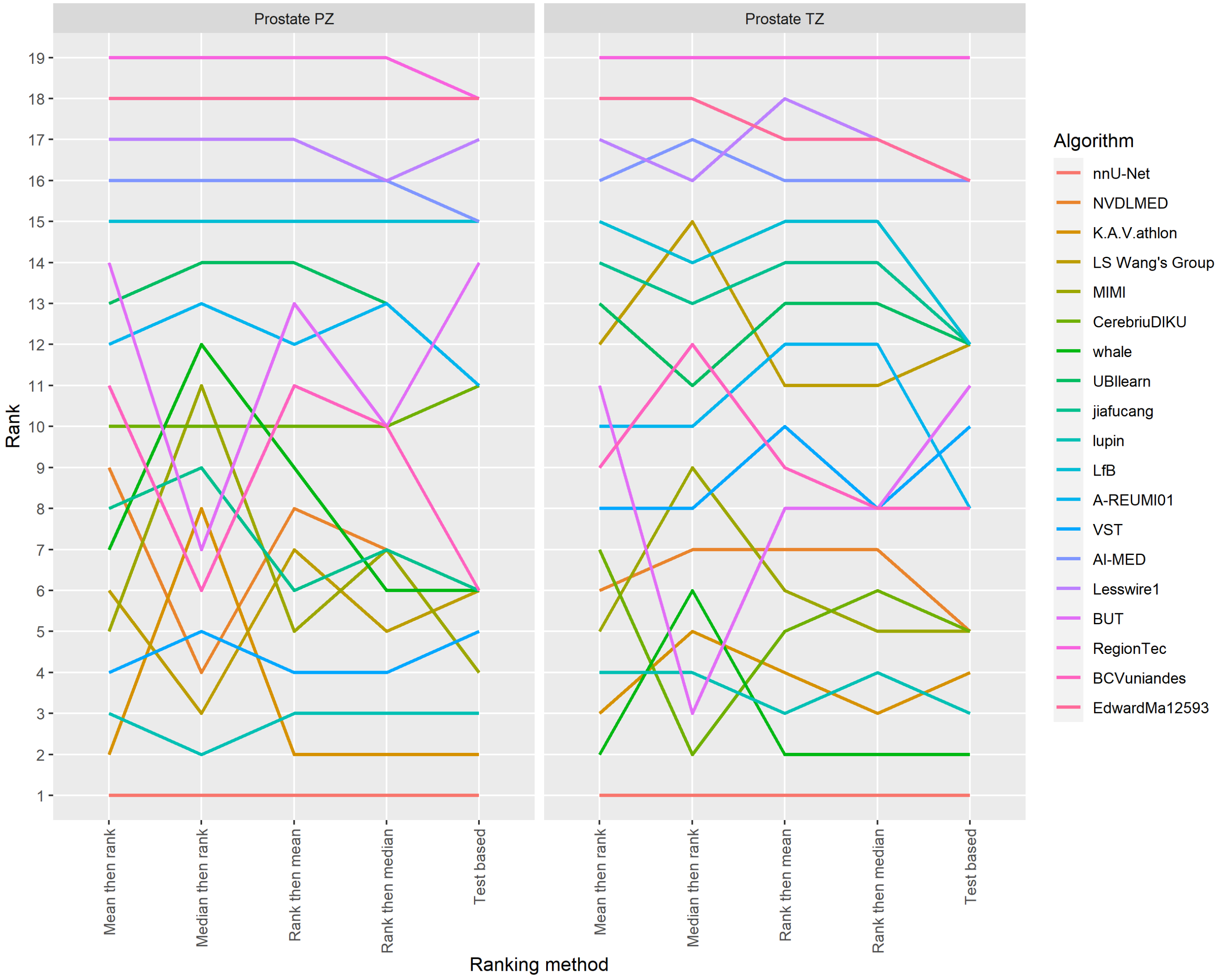

Supplementary Figure 9: Line plots visualizing rankings robustness across four different ranking methods for the prostate task. Each of the 19 algorithms is represented by one colored line. For every ranking method encoded on the x-axis, the height of the line represents the corresponding rank. Horizontal lines indicate identical ranks for all methods under all ranking criteria.

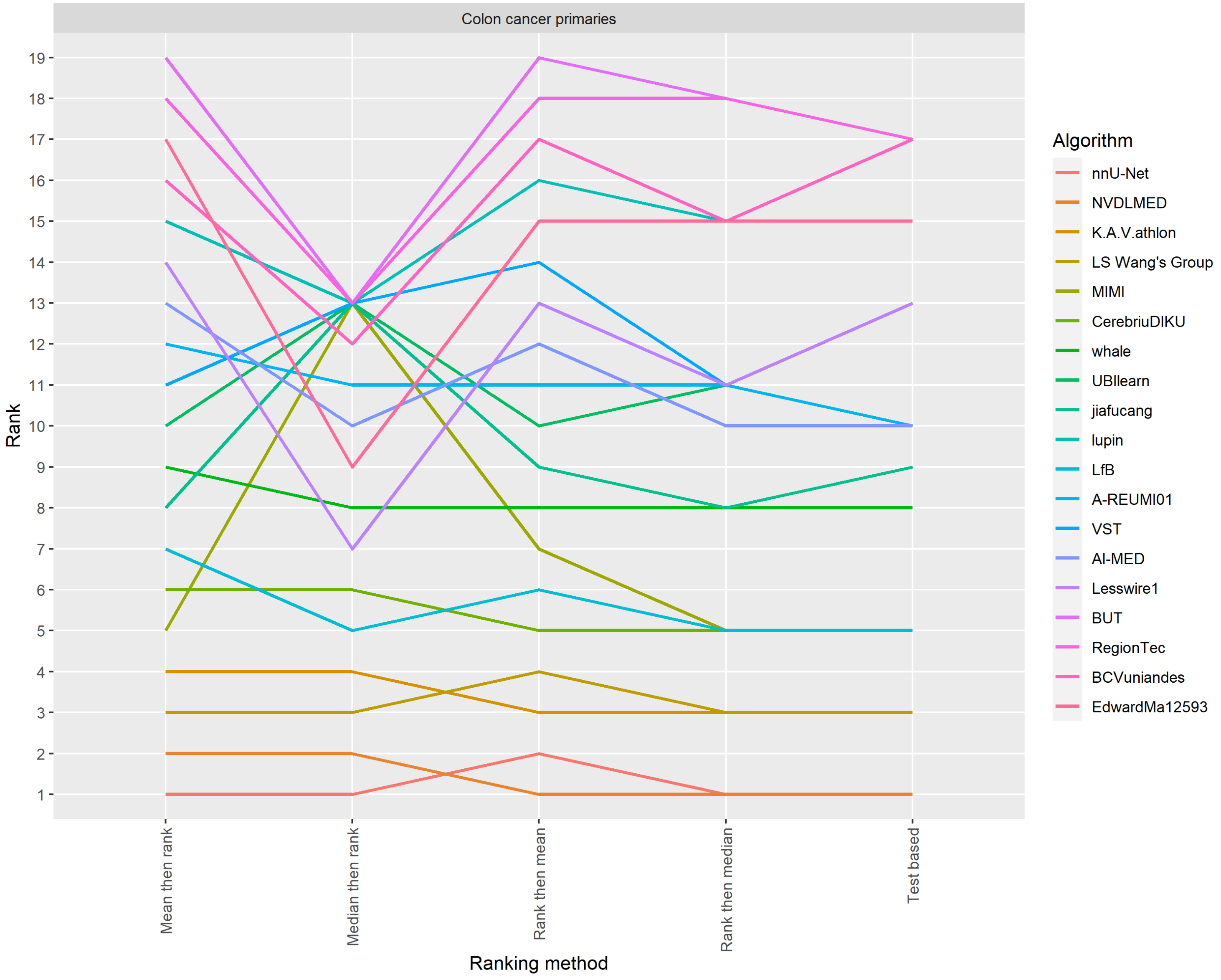

Supplementary Figure 10: Line plots visualizing rankings robustness across four different ranking methods for the colon task. Each of the 19 algorithms is represented by one colored line. For every ranking method encoded on the x-axis, the height of the line represents the corresponding rank. Horizontal lines indicate identical ranks for all methods under all ranking criteria.

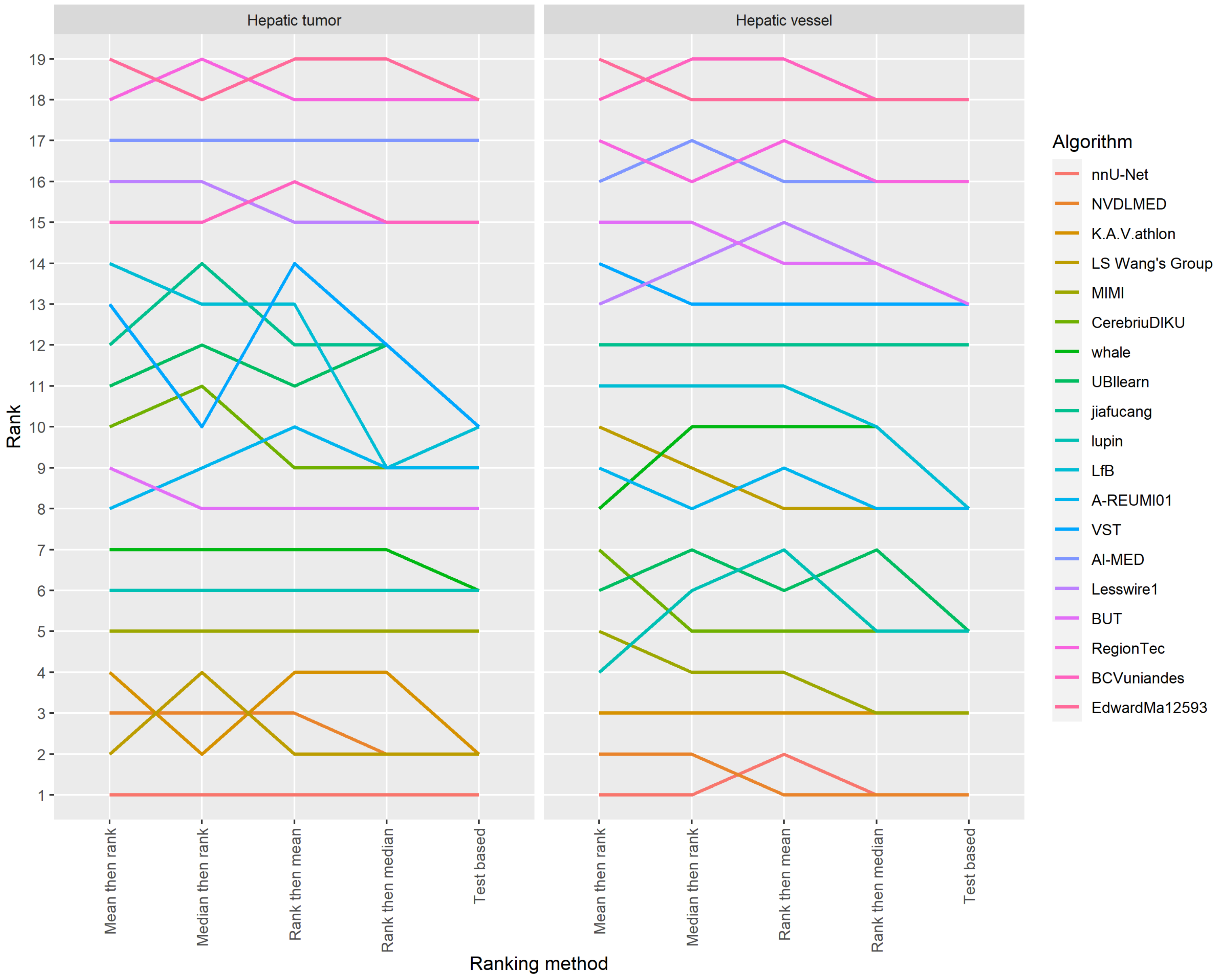

Supplementary Figure 11: Line plots visualizing rankings robustness across four different ranking methods for the hepatic vessel task. Each of the 19 algorithms is represented by one colored line. For every ranking method encoded on the x-axis, the height of the line represents the corresponding rank. Horizontal lines indicate identical ranks for all methods under all ranking criteria.

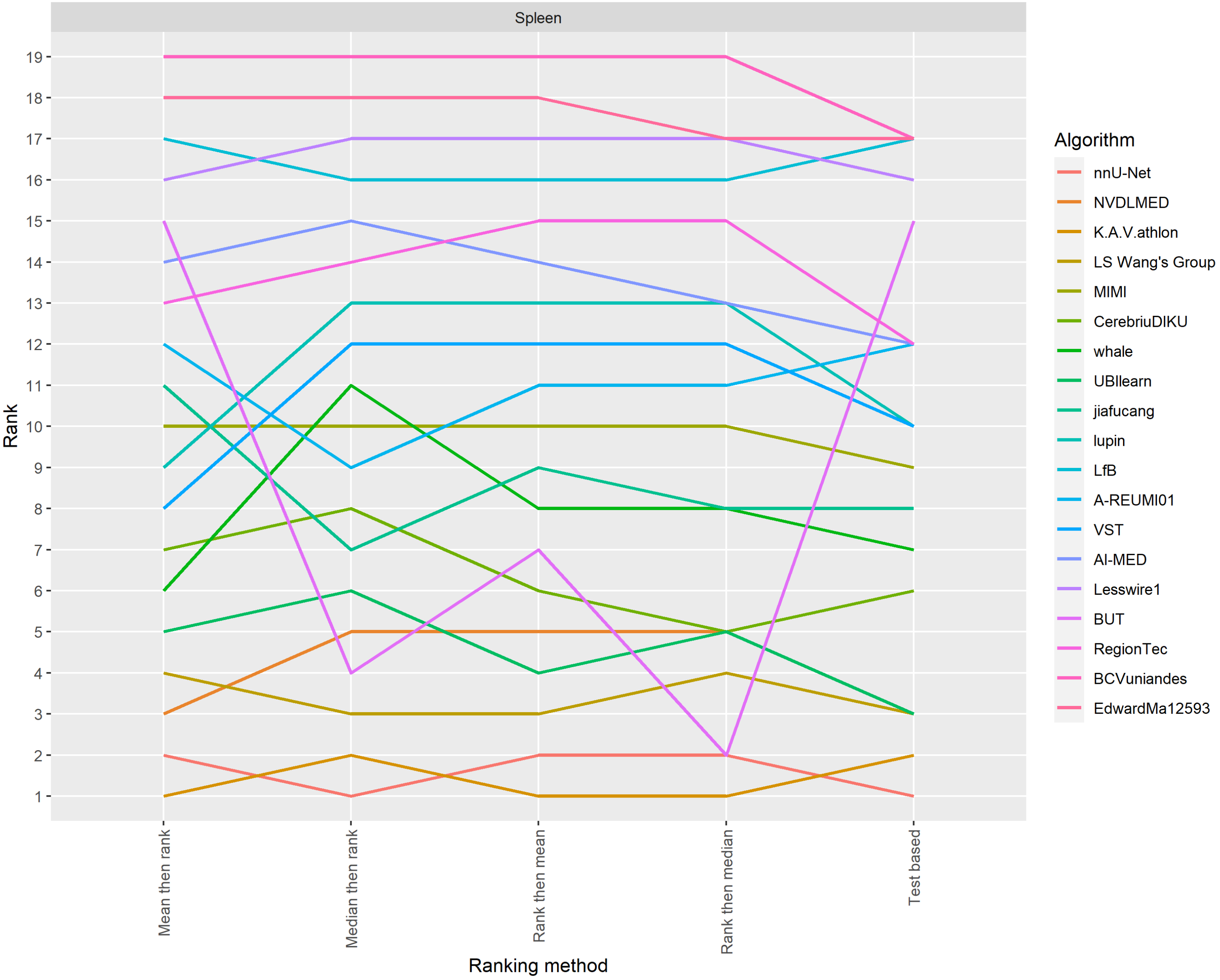

Supplementary Figure 12: Line plots visualizing rankings robustness across four different ranking methods for the spleen task. Each of the 19 algorithms is represented by one colored line. For every ranking method encoded on the x-axis, the height of the line represents the corresponding rank. Horizontal lines indicate identical ranks for all methods under all ranking criteria.

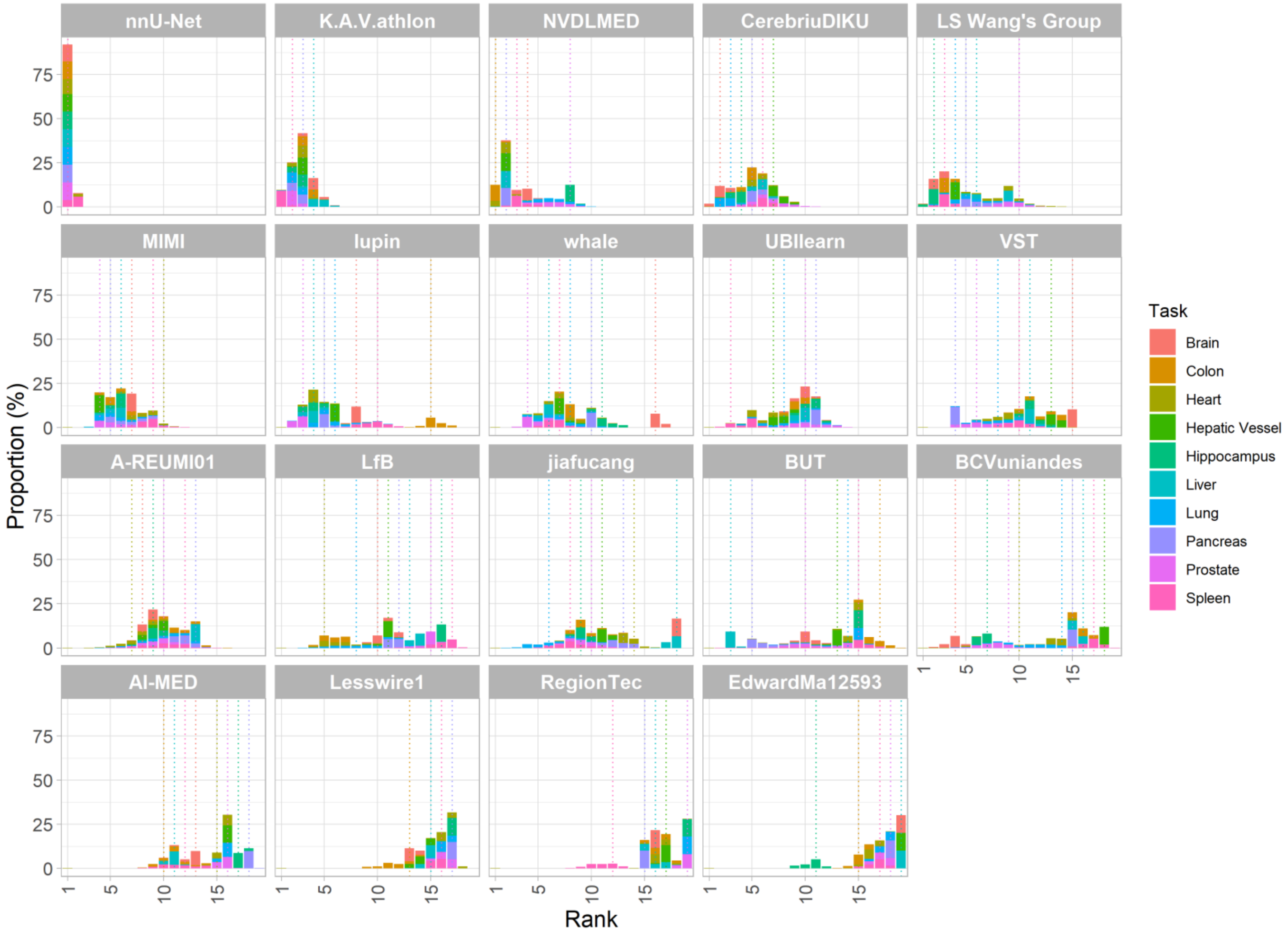

Supplementary Figure 13: Stacked frequency plot showing the achieved ranks of the 19 participating algorithms over 1,000 bootstrap datasets for all tasks (color-coded) for the DSC. Vertical lines indicate algorithms that achieved the same rank for the whole data set. The plot was created using challengeR.

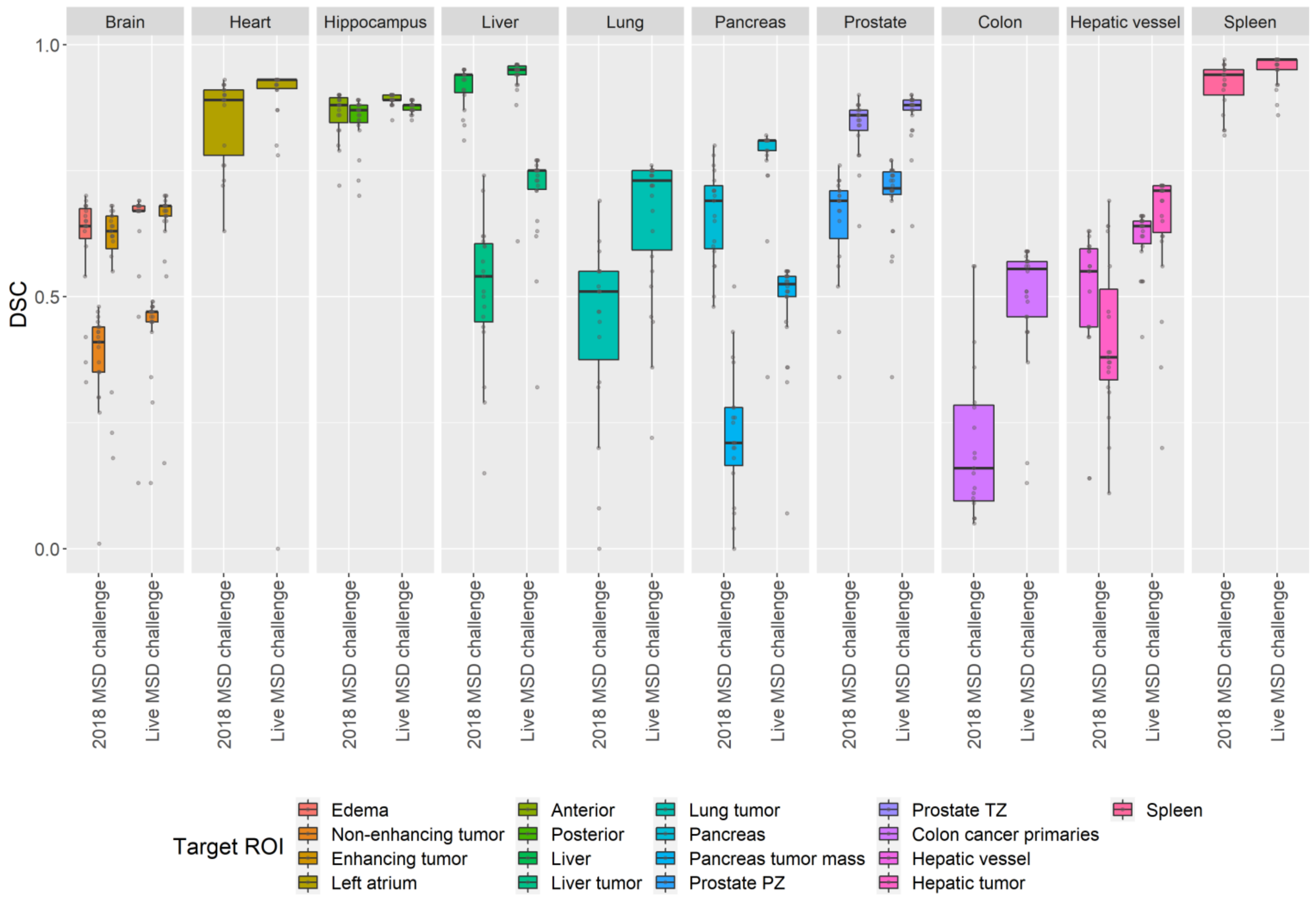

Supplementary Figure 14: Dot- and box-plot of the mean DSC values computed for each task and target ROI for all algorithms in the 2018 MSD (n = 19 teams) and live-decathlon (n = 26 teams) challenges. box-plots represent descriptive statistics over all mean DSC values of each participant. The median value is shown by the black horizontal line within the box, the first and third quartiles as the lower and upper border of the box, respectively, and the 1.5 interquartile range by the vertical black lines. The mean DSC values per participant are provided as gray circles.

References

[1] Roy, A.G., Conjeti, S., Navab, N., Wachinger, C., 2018. Quicknat: Segmenting MRI neuroanatomy in 20 seconds. CoRR abs/1801.04161. URL: http://arxiv.org/abs/1801.04161.

[2] Kamnitsas, K., Ferrante, E., Parisot, S., Ledig, C., Nori, A.V., Criminisi, A., Rueckert, D., Glocker, B., 2016. Deepmedic for brain tumor segmentation, in: International workshop on Brainlesion: Glioma, multiple sclerosis, stroke and traumatic brain injuries, Springer. pp. 138–149.

[3] Perslev, M., Dam, E.B., Pai, A., Igel, C., 2019. One network to segment them all: A general, lightweight system for accurate 3d medical image segmentation, in: International Conference on Medical Image Computing and Computer-Assisted Intervention, Springer. pp. 30–38.

[4] Rippel, O., Weninger, L., Merhof, D., 2020. Automl segmentation for 3d medical image data: Contribution to the msd challenge 2018. URL: https://arxiv.org/abs/2005.09978.

[5] Wang, L., Chen, R., Wang, S., Zeng, N., Huang, X., Liu, C., 2019. Nested dilation network (ndn) for multi-task medical image segmentation. IEEE Access 7, 44676–44685.
