# Supplementary material for: The Medical Segmentation Decathlon
Source: Nat Commun. 2022 Jul 15;13:4128. doi: 10.1038/s41467-022-30695-9 (PMC9287542; doi:10.1038/s41467-022-30695-9)
Supplement: Supplementary file 2 — Reporting Summary [file 41467_2022_30695_MOESM2_ESM.pdf]

Corresponding author(s): *Double-blind peer review submissions: write DBPR and your manuscript number here instead of author names.*

Last updated by author(s): *YYYY-MM-DD*

Reporting Summary

Nature Portfolio wishes to improve the reproducibility of the work that we publish. This form provides structure for consistency and transparency in reporting. For further information on Nature Portfolio policies, see our Editorial Policies and the Editorial Policy Checklist.

Statistics

For all statistical analyses, confirm that the following items are present in the figure legend, table legend, main text, or Methods section.

n/a	Confirmed	
☐	☒	The exact sample size (n) for each experimental group/condition, given as a discrete number and unit of measurement
☐	☐	A statement on whether measurements were taken from distinct samples or whether the same sample was measured repeatedly
☐	☒	The statistical test(s) used AND whether they are one- or two-sided<br>*Only common tests should be described solely by name; describe more complex techniques in the Methods section.*
☐	☒	A description of all covariates tested
☒	☐	A description of any assumptions or corrections, such as tests of normality and adjustment for multiple comparisons
☐	☒	A full description of the statistical parameters including central tendency (e.g. means) or other basic estimates (e.g. regression coefficient) AND variation (e.g. standard deviation) or associated estimates of uncertainty (e.g. confidence intervals)
☒	☐	For null hypothesis testing, the test statistic (e.g. F, t, r) with confidence intervals, effect sizes, degrees of freedom and P value noted<br>*Give P values as exact values whenever suitable.*
☒	☐	For Bayesian analysis, information on the choice of priors and Markov chain Monte Carlo settings
☐	☒	For hierarchical and complex designs, identification of the appropriate level for tests and full reporting of outcomes
☒	☐	Estimates of effect sizes (e.g. Cohen's d, Pearson's r), indicating how they were calculated

Our web collection on statistics for biologists contains articles on many of the points above.

Software and code

Policy information about availability of computer code

Data collection	The images (2,633 in total) were acquired across multiple institutions, anatomies and modalities during real-world clinical applications. All images were de-identified and reformatted to the Neuroimaging Informatics Technology Initiative (NIfTI) format.<br><br>The challenge assessment data (results from challenge participants) has been collected by computing the DSC and NSD values based on the submitted methods compared to the reference annotations.
Data analysis	Metric values (DSC and NSD) have been computed based on the submitted algorithm results and the reference annotations. For this purpose, a Python script was utilized: http://medicaldecathlon.com/files/Surface_distance_based_measures.ipynb<br><br>The data was analyzed with R (version 4.1.0). For the ranking (uncertainty) analysis, the package challengeR (version 1.0.2; https://github.com/wiesenfa/challengeR) and the IDE RStudio were used.

For manuscripts utilizing custom algorithms or software that are central to the research but not yet described in published literature, software must be made available to editors and reviewers. We strongly encourage code deposition in a community repository (e.g. GitHub). See the Nature Portfolio guidelines for submitting code & software for further information.

Data

Policy information about availability of data

All manuscripts must include a data availability statement. This statement should provide the following information, where applicable:

- Accession codes, unique identifiers, or web links for publicly available datasets
- A description of any restrictions on data availability
- For clinical datasets or third party data, please ensure that the statement adheres to our policy

Challenge data set: The MSD data set is publicly available under a Creative Commons license CC-BY-SA4.0, allowing broad (including commercial) use. The training data used in this study is available at http://medicaldecathlon.com/. The test data of the challenge cannot be released since the live challenge is still open.

Challenge assessment data: The raw challenge assessment data used to calculate the challenge rankings can not be made publicly available due to privacy reasons. It contains the DSC and NSD values for every participating team for every task and target region. However, the aggregated results can be found in Table 2 and Tables D.4-D.13. Furthermore, they can be found here: https://phabricator.mitk.org/source/msd_evaluation/ in the folders descriptive-statistics, mean-values-per-subtask and rankings-per-subtask.

Field-specific reporting

Please select the one below that is the best fit for your research. If you are not sure, read the appropriate sections before making your selection.

[x] Life sciences [ ] Behavioural & social sciences [ ] Ecological, evolutionary & environmental sciences

For a reference copy of the document with all sections, see nature.com/documents/nr-reporting-summary-flat.pdf

Life sciences study design

All studies must disclose on these points even when the disclosure is negative.

Sample size	No sample-size calculation was performed. For the imaging data, we collected as much data as possible to enable the machine learning methods to be able to train properly. For the challenge assessment data, sample size was restricted to the number of participating algorithms who submitted valid results (n = 19).
Data exclusions	No data was excluded.
Replication	All results were reproduced internally based on this repository: https://phabricator.mitk.org/source/msd_evaluation/.
Randomization	Train/test data was split random for all datasets except BRATS where we were asked to preserve the same partition used in the BRATS challenge
Blinding	All challenge participants and organisers (except the first and last author) have no access to the challenge ground truth. All challenge results were analysed automatically using a pre-defined/agreed script and methodology, thus blinding was deemed unnecessary.

Reporting for specific materials, systems and methods

We require information from authors about some types of materials, experimental systems and methods used in many studies. Here, indicate whether each material, system or method listed is relevant to your study. If you are not sure if a list item applies to your research, read the appropriate section before selecting a response.

Materials & experimental systems

n/a	Involved in the study	
x		Antibodies
x		Eukaryotic cell lines
x		Palaeontology and archaeology
x		Animals and other organisms
	x	Human research participants
x		Clinical data
x		Dual use research of concern

Methods

n/a	Involved in the study	
x		ChIP-seq
x		Flow cytometry
x		MRI-based neuroimaging

Human research participants

Policy information about studies involving human research participants

Population characteristics	No detailed information for the challenge imaging data was available, as the data set connects data from many different centers from anoymized patientes.<br>Brain data: Patients diagnosed with either glioblastoma or lower-grade glioma.<br>Heart data: Patients undergoing mono-modal MRI scans of the entire heart acquired during a single cardiac phase (free breathing with respiratory and electrocardiogram (ECG) gating)<br>Hippocampus data: MRI images acquired from healthy adults and adults with a non-affective psychotic disorder<br>Liver data: Patients with primary cancers and metastatic liver disease, as a consequence of colorectal, breast, and lung primary cancer.<br>Lung data: Patients with non-small cell lung cancer.<br>Prostate data: Probands from multiparametric MRI (mpMRI) studies<br>Pancreas data: Patients undergoing resection of pancreatic masses<br>Colon data: Patients undergoing resection of primary colon cancer<br>Hepatic vessel data: Patients with a variety of primary and metastatic liver tumors<br>Spleen data: Patients undergoing chemotherapy treatment for liver metastases
Recruitment	All datasets used in this work were re purposed from other investigational studies, and thus constitute secondary use of the data. All patients were originally recruited following standard IRB/REC processes
Ethics oversight	All datasets have been approved by Internal Review Boards or Research Ethics Committees as described in detail in https://arxiv.org/abs/1902.09063

Note that full information on the approval of the study protocol must also be provided in the manuscript.
